# Wildfire and asthma - The prospective interventions^☆^

**DOI:** 10.1016/j.waojou.2025.101110

**Published:** 2025-09-03

**Authors:** Xingxing Yuan, Liuxin Yang, Chaofan Li, Ousman Bajinka, Zhuying Li

**Affiliations:** a First Clinical Medical College, Heilongjiang University of Chinese Medicine, Harbin 150040, China; b Department of Gastroenterology, Heilongjiang Academy of Traditional Chinese Medicine, Harbin 150006, China; c Medical Science and Technology Innovation Center, Shandong Key Laboratory of Oncology, Shandong Cancer Hospital and Institute, Shandong First Medical University and Shandong Academy of Medical Sciences, China; d Department of Respiratory, First Affiliated Hospital of Heilongjiang University of Chinese Medicine, Harbin, China

**Keywords:** Asthma, Wildfire, Particulate matter, Respiratory tract diseases, Public health

## Abstract

The increasing global warming trend has led to a drier landscape, which has in turn resulted in an increased incidence of wildfires. The smoke emanating from these fires has consequences that extend beyond the loss of property. This comes with the wildfire season whose smoke emanations have implications beyond loss of property. Among the health concerns regarding wildfires and smoke are respiratory diseases, such as asthma. Asthma, as a heterogeneous disease, is directly linked to other types of smoke rather than cigarettes, traffic exhaust, and industrial chemicals. Wildfires have been shown to be associated with particulate matter that act as pollutants to human life. The unprecedented increase in emergency visits during wildfire seasons is of clinical significance for its association with asthma and other pulmonary disorders. Despite the efforts of wealthy communities or nations, which have faced episodes of wildfires in recent years, the most effective protocol is yet to be developed. Given the vulnerability of individuals, including asthmatics, to the smoke from wildfires, interventions should extend beyond emergency measures. This review aims to provide a comprehensive overview of the relationship between wildfires and asthma symptoms, exploring the underlying mechanisms both *in vitro* and *in vivo*. It also delves into the potential implications for science policies, communication strategies, research directions, and management strategies for asthma cases, emphasizing the importance of preventive measures. Furthermore, this review serves as a guideline for various sectors, offering a clear conceptual rationale for preventing and managing wildfire smoke-related asthma and other pulmonary diseases. The interventions are multi-sectoral and multidisciplinary concerns, and given the transboundary nature of smoke, it is imperative for all relevant parties to collaborate to minimize preventable deaths.

## Introduction

Asthma, a heterogeneous chronic airway disease, is strongly associated with elevated cardiopulmonary mortality risks during wildfire events.^1^^,^^2^ While wildfires naturally contribute to terrestrial ecological cycles, their emissions—particularly particulate matter 2.5/10 (PM2.5/10), nitrogen oxides, carbon monoxide, and volatile organic compounds—pose significant threats to public health and economies.^3^, ^4^, ^5^ Climate change, driven by fossil fuel use, exacerbates wildfire frequency through extreme heat and prolonged droughts. These conditions intensify air pollution via wildfire-derived PM and ozone precursors, the latter of which aggravates childhood asthma symptoms during haze episodes.^6^ Although individuals with healthy respiratory systems may experience milder acute effects, those with pre-existing conditions (eg, asthma, chronic obstructive pulmonary disease, chronic sinusitis) face disproportionately higher risks.^7^, ^8^, ^9^ Wildfire smoke exposure is also linked to systemic health impacts, including cardiovascular diseases (ischemic heart disease, acute myocardial infarction),^10^, ^11^, ^12^ sleep disorders,^13^ and mental health exacerbations (eg, schizophrenia symptom severity),^14^ potentially mediated by inflammatory responses to pollutants.

Global warming contributes to a warmer climate, thereby exacerbating dry conditions and fueling the spread of wildfires. These wildfires have been shown to cause building damage, property loss, and loss of life. Moreover, they have a profound impact on human health. For example, exposure to heat waves caused by wildfires can lead to physical and emotional harm on humans.^15^ Additionally, greenhouse gases (GHGs) released by wildfires can exacerbate pollen allergies and asthma.^16^ Furthermore, PM2.5, a significant air pollutant derived from wildfires, has been associated with an elevated prevalence of asthma, emergency department visits, and hospitalizations.^17^^,^^18^ Additionally, PM2.5 has been implicated in the induction of local and systemic inflammation, oxidative stress, and airway epithelial damage, thereby increasing the risk of infection.^19^ Children and young adults are particularly vulnerable to symptoms such as dyspnea, wheezing, and cough.^20^^,^^21^ As the global population grows, human activities are directly linked to climate change, which can further exacerbate dry environments and lead to increased wildfires.^22^ The validity of these health effects has been substantiated by pertinent cases from the British Columbia Asthma Prediction System (BCAPS) and the Asthma Healthcare Utilization (AHCU).^23^

The increasing trend of landscape fires driven by climatic events has profoundly impacted human health and socio-economic activities in many countries. Health organizations and public health agencies are currently developing emergency protocols, particularly plans to evacuate high-risk populations during wildfire smoke events.^24^, ^25^, ^26^, ^27^ Research indicates that children and adolescents, due to their high metabolic rates, narrow airways, and ongoing physical development, are more susceptible to respiratory symptoms and require increased medical attention.^28^ Additionally, firefighters and individuals with no prior respiratory disease history may also experience irreversible respiratory damage from wildfire smoke exposure.^29^^,^^30^ Notably, respiratory issues such as asthma often peak 1 year after wildfire smoke exposure, potentially linked to delayed immune responses triggered by allergic sensitization.^31^ Beyond wildfire smoke, extreme weather events like tropical cyclones and thunderstorms may exacerbate asthma symptoms by worsening air pollution or altering temperature and humidity. Consequently, community-based health education is critical to promote simple risk-mitigation measures.^32^ This review aims to explore prospective interventions for the prevention of asthma associated with wildfire exposure and to elucidate the mechanistic pathways underlying the impact of wildfire smoke on asthma.

### Wildfire-induced mechanisms of asthma

As the upper respiratory tract serves as the primary interface between the external environment and the human body, it is highly susceptible to damage from air pollution, including wildfire smoke.^33^ Rising surface temperatures, which exacerbate wildfire frequency, exert both direct and indirect effects on allergic-immunologic diseases through cellular and molecular pathways.^34^ Wildfire smoke disrupts epithelial integrity, thereby impairing immune regulation and promoting systemic inflammation. Advanced ground-level monitoring combined with spatiotemporal modeling may obstruct this chain.^35^ Climate change exacerbates health risks for individuals with asthma and allergic-immunologic conditions through multiple pathways, notably by increasing exposure to respiratory triggers such as atmospheric allergens and ground-level ozone.^36^ Furthermore, PM mediates variations in susceptibility to climate-sensitive diseases among community and prenatal populations by inducing epigenetic alterations in DNA methylation.^37^^,^^38^ This exposure-induced epigenetic reprogramming offers molecular evidence for environment-gene interactions.

**Fig. 1:**
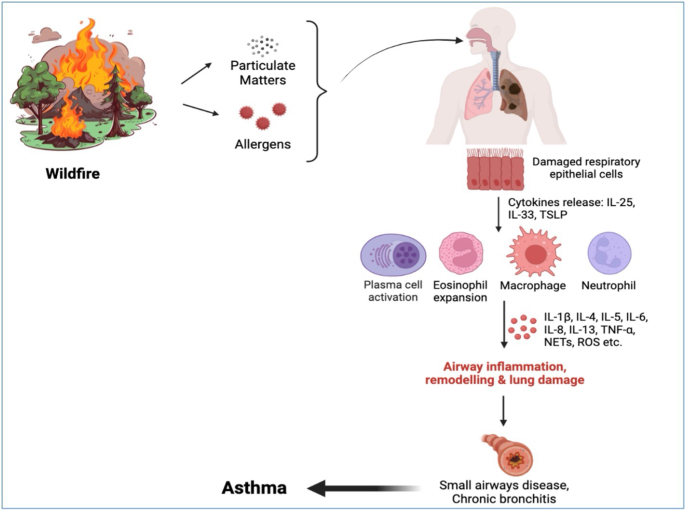
Mechanism of wildfire-induced asthma.

Notably, woodsmoke-derived PM alters gene expression in the airways and increases susceptibility to respiratory viral infection, which in turn resulted in more frequent asthma attacks and exacerbations.^39^ The disruption of structural proteins invariably leads to airway inflammation and hyperactivity. This, in turn, has a subsequent effect on the thermoregulatory system, resulting in increased respiratory rate and tidal volume. Moreover, reflex bronchoconstriction and specific airway resistance result in increased transient receptor potential vanilloid 1/4 (TRPV1/4) and bronchopulmonary vagal C-fibers activation.^40^ Mechanistically, wildfire smoke exacerbates these effects via 3 synergistic pathways: eliciting systemic inflammation, inducing oxidative stress, and activating xenobiotic response systems.^41^^,^^42^ Cellular-level analyses reveal that prolonged smoke exposure dysregulates alveolar epithelial function via MAPK signaling pathway impairment.^43^ This exposure duration correlates with marked upregulation of immune mediators, including killer cell immunoglobulin-like receptor 3DL1(KIR3DL1), granzymes A, B, and H (GzmA/B/H), IL-16, nibrin (NBN), C1q TNF-related protein (CTRP), poly (ADP-ribose) polymerase 1 (PARP1), and fibroblast growth factor 19 (FGF19).^44^ These coordinated changes demonstrate significant functional alterations in natural killer cells and associated immune networks, establishing a mechanistic link between smoke exposure and compromised antiviral defenses. Table 1 and Fig. 1.

**Table 1:** Clinical trials on asthma episodes and management

Objective	Study design	Main findings	Conclusions	Reference
▪ Anti-inflammatory and antioxidant effects of N. officinale hydroalcoholic extract (NOE)	▪ Randomized, double-blind, and placebo-controlled trial ➢ Asthma Control Test score	▪ Reduction in the levels of MDA, PCO and NO metabolite markers after 1 month ▪ FRAP levels significantly increased at the end of the treatment period	▪ NOE may have a therapeutic effect on asthma by improving oxidative stress	45
▪ Association of lung function deficits with inflammatory cytokines ▪ Children with acute chest syndrome (ACS)	▪ Two-year RCT ➢ Serum cytokines ➢ Leukotriene B4 levels ➢ Pulmonary function tests (PFTs)	▪ Lower total lung capacity (TLC) ▪ IL-5, and IL-13 were higher at baseline and at 2 years ▪ IP-10 and IL-6 were negatively correlated with PFT markers ▪ Asthma status was associated with FEV1	▪ Airway inflammation present in children with SCD and ACS could impair pulmonary function	46
▪ Implementation of the coach McLungs^SM^ intervention into primary care ▪ Shared decision making (SDM) intervention	▪ RCT ➢ Generalized linear mixed models with logit link ▪ EDVs ▪ Hospitalizations ▪ Oral steroid use	▪ Implementations to be used ➢ Expert Recommendations for Implementing Change (ERIC) ➢ CFIR (consolidated Framework for Implementation Research) ➢ RE-AIM (Reach Effectiveness, Adoption, Implementation, maintenance).	▪ A decrease EDVs, hospitalizations, and oral steroid use for asthmatic patients is anticipated	47
▪ Test-and-treat approach to correction of suboptimal vitamin D ▪ Acute respiratory tract infection	▪ Phase 3 open label RCT ➢ Six-month supply of vitamin D ➢ Postal finger prick test of blood	▪ Acceptance rate is 95.4% ▪ At least 1 acute respiratory tract infection of any cause occurred in 5.7%	▪ For suboptimal vitamin D status, this intervention can help reduce the risk of acute respiratory tract infection	48
▪ Counseling approaches ▪ Asthmatic children who use metered-dose inhaler (MDI)	▪ RCT ➢ mistakes in inhalation technique steps ➢ Detected, corrected, and recorded and the inhalation duration was measured	▪ A significant reduction in the total mean number of mistakes and a significant increase in total mean inhalation durations were observed	▪ A combination counseling traditional verbal and advanced counseling may lead to improvements in the number of inhalation technique mistakes and inhalation durations	49
▪ Inhaled budesonide-formoterol inhaler, ▪ Chronic respiratory diseases	▪ RCT ➢ Symptoms ➢ maintenance plus as-needed ➢ Referral	▪ Diagnosed with an exacerbation (35%) ▪ Adhered to inhaled budesonide-formoterol after 12 months (50%)	▪ Therapeutic algorithm is feasible	50
▪ Dietary Approaches to Stop Hypertension (DASH) diet and asthma pathophysiology	▪ RCT –exploratory ➢ Serum proteins ➢ Biomarkers of asthma	▪ Changes in IL-1β, transforming growth factor α (TGF-α), and IL-6 ▪ Asthmatic conditions, specifically several T-helper (Th) 2 and Th17 ▪ Improved asthma control	▪ Introduction of DASH may reduce inflammatory status in adult asthmatics	51
▪ BREATHE (BRief intervention to Evaluate Asthma THErapy), ▪ SDM intervention	▪ Group-randomized longitudinal pilot study ➢ Surveys ➢ lung function tests ➢ Interviews	▪ Better asthma control at each time point ▪ Perceived greater SDM and fewer symptoms at follow-up	▪ This can be integrated into office visits using clinicians as interventionists	52
▪ Asthma management in pregnancy ▪ Treatment decision differences	▪ RCT ➢ baseline blood eosinophils	▪ Increased in eosinophilic asthma ▪ Fewer women had exacerbations during pregnancy	▪ This algorithm is effective in treating asthma exacerbations among pregnant women with asthma.	53
▪ Nonadherence in adults with asthma comprehensibility, coherence and acceptability.	▪ Survey and interviews ➢ Person-based, qualitative approach to investigate	▪ Logical and easily understandable	▪ Patient-based approaches may facilitate implementation and acceptability of interventions in practice	54
▪ Asthma SDM intervention into primary care practices	▪ RCT ➢ A facilitator-led approach ➢ A 1-h lunch-and-learn training ➢ A one-question anonymous survey	▪ More likely to report that they participated equally ▪ No significant differences for ED visits, hospitalizations or oral steroids	▪ The use of structured approaches is promising asthma treatment approach	55

## Prospective interventions against wildfire-induced asthma

### Policy interventions

Proximity to wildfires is associated with increased susceptibility to respiratory infections, including asthma.^56^ However, existing data remain insufficient to inform robust policy decisions.^57^ To effectively mitigate the health impacts of wildfires, a multi-faceted approach is essential. Reduction in carbon emissions and revisiting climate change and health policies can play a crucial role. Specifically, these measures can slow down the frequency and intensity of wildfires, thereby providing a grace period during which we can better prepare for and prevent the severe impacts of wildfires on human health.^58^ Although there are already established policies aimed at curbing the worsening wildfire situations, these policies may have limitations due to the lack of sufficient data. Forecast-based interventions can complement these existing policies, such as conservation efforts,^59^ advance research on wildfire smoke forecasting systems,^60^ and considering air quality, public health interests, fire safety, and environmental justice will lead to trans-disciplinary discussions.^61^

Social disadvantages communities demonstrate heightened vulnerability to wildfire smoke due to intersecting risk factors including ethnic minority status, crowded housing, low educational attainment, inadequate healthcare access, and food/job insecurity.^62^^,^^63^ These compounding disparities necessitate prioritized public health interventions. Current initiatives aim to highlight these inequities, implement activity restrictions during smoke events, and enhance asthma monitoring throughout the wildfire season. However, deploying asthma self-management technologies—particularly mobile applications proven to reduce risks among youth—establishing federal response (including fire behavior prediction systems, real-time air quality modeling, emergency clinical communication platforms, and health impact assessment frameworks), could mitigate wildfire-related health impacts.^64^, ^65^, ^66^ At the household level, air filtration systems and clean-air shelters with effective public health messaging are pivotal preventive policy.^67^ This tiered approach addresses both systemic vulnerabilities and immediate protective needs during wildfire events.

Although PM10 concentration remains a significant predictor of asthma emergency department visits, healthcare organizations, other respiratory tract infections as comorbidities, and patient self-control and compliance should form an integral part of the One Health approach for asthma care.^68^ Educational initiatives enhancing self-care competence have been shown to generally improve symptom control, although the effectiveness of these initiatives is diminished when addressing non-adherent patient populations.^69^ Furthermore, the implementation of personalized care for severe asthmatics is non-linear and complex.^70^ To mitigate this, besides the strict adherence, high levels of physical activity for adult asthmatic patients and elementary school-based exercise can boost the immune system.^71^^,^^72^ For the pediatric population, particularly those from low-income backgrounds, structured programs such as Complex Children's Healthcare Coordination (CHECK) have been shown to be effective in controlling prescriptions and enhancing healthcare utilization.^73^ Clinicians who are the first to detect climate change related diseases should urgently inform epidemiologists who will effectively source track and establish the disease chain. Likewise, this chain must urgently find its way to influencing policy.^74^

### Science communication

School curricula should integrate wildfire smoke education modules to introduce the health risks of particulate matter exposure, environmental warning signals, and age-appropriate evacuation protocols.^75^ District-level implementation requires certified pediatric respiratory specialists to conduct biannual training simulations, ensuring alignment with local emergency response frameworks.^76^^,^^77^ Allergology practitioners must prioritize air quality counseling during peak wildfire seasons, emphasizing preventive pharmacotherapy adjustments and caregiver protective measures.^78^

Strategic public health communication and engagements in protective behavior adaptation or behavioral mediation, and taking measures against dangers associated with natural disasters, should be effectively implemented.^79^, ^80^, ^81^ Community-based care initiatives have shown promise in reducing asthma-related healthcare burdens, offering localized solutions to manage symptoms more effectively.^82^ However, a critical global gap remains in health literacy for self-management during emergencies, as preventable deaths persist when supportive interventions are not adequately understood or accessible.^83^ A multifaceted, comprehensive approach has been demonstrated to be effective in maintaining asthma conditions through collaborative learning and adherence to hospital guidelines.^84^ The novel SARS-CoV-2 virus of 2019 and COVID-19 disease pandemic prompted behavioral changes, such as mask-wearing. While it may not impact evacuation from wildfires,^85^ this healthcare precaution correlates with a decrease in asthma exacerbations, underscoring the importance of masks in combating air pollution, which is a major contributing factor to asthma.^86^ Consequently, integrated asthma counseling is imperative for effective health and science communication interventions.^87^

### Research priorities

Given the established risks of wildfire smoke exposure to allergic airway sensitization and asthma exacerbations, research should prioritize biomarker discovery for severe exacerbation prediction and prevention. Retrospective cohort analyses of relocated populations could prospectively evaluate long-term health impacts, as improved living conditions may lead to underreporting of childhood or chronic exposure histories in predisposed individuals.^88^ Apparently, there is small but growing evidence linking exposure to wildfires to early life respiratory health. This evidence underscores the need for further research, including large cohort studies, to establish evidence-based medicine.^89^

Epidemiological investigations should employ concentration-response functions (CRFs) with multi-source datasets to quantify asthma burden and other wildfire-related health impacts.^90^ Such investigations should not be limited to exposed individuals; they should also consider maternal baseline exposure. Adverse birth outcomes, such as preterm birth and low birth weight, are associated with respiratory distress in offspring, which is epigenetically linked to early life. To this end, the need for advanced biological pathways is imperative to establish the detailed adverse health outcomes.^91^ Elucidating the relationship between prenatal stress and wildfires could facilitate the generation of evidence-based findings on offspring's early childhood respiratory health outcomes, as evidenced by a substantial cohort employing appropriate study designs.^92^ Maternal antibiotic use and its association with childhood asthma can act as confounding factors in pediatric asthma research.^93^ The hygiene hypothesis posits that less socialized children exhibit a heightened susceptibility to asthma compared to those who are more socialized, particularly in the context of pet ownership, such as dogs.^94^

In asthma clinical trials, the recruitment process could bring in confounding factors. For instance, Sublingual immunotherapy (SLIT) can lower asthma attacks only for those with mild and intermittent asthma exacerbations.^95^^,^^96^ For that reason, studies should also focus on severe asthma cases. For epigenetically linked studies on mtDNA and bushfire-related PM, bedroom dust allergens and indoor PM must not make confounding factors.^97^ Machine-learning algorithms and artificial intelligence (AI) applications, such as Engulfed Carbon Particles (MacLEAP), hold great promise in comprehensively addressing the associated respiratory distress.^98^ Single-cell RNA sequencing with spatial data is essential for the Human Lung Cell Atlas (HLCA) to serve as a reference. This will establish the foundation for immune-regulatory mechanisms studies and facilitate evidence-based translational studies.^99^ Mixtures-based approaches are crucial to identify the potential drivers of wildfire exposure,^67^ and effective smoke exposure estimation methods are required to validate for standards.^100^

### Asthma case management

Like lung cancer, the detrimental effects of wildfire-induced asthma often manifest only after significant damage has already occurred. This underscores the critical importance of early recognition and intervention. It is essential to distinguish these effects from those caused by traffic-related, industrial, or cigarette smoke. In this context, advancements in respiratory medicine may require more sophisticated technologies that go beyond detecting lung inflammation, potentially incorporating principles from the germ theory of diseases.^101^ To this end, firefighters frequently exposed to wildfire smoke could participate in large-scale cohort studies aimed at understanding immune system alterations due to such exposures.^67^ Additionally, there is an urgent need to establish emergency healthcare setups for rural populations affected by wildfire smoke, particularly during wildfire seasons.^102^ The use of rescue medications for older children could be life-saving.^103^ Furthermore, preventive medicine strategies should focus on minimizing the forced vital capacity in childhood asthma patients while conducting extensive studies on climate change patterns.^44^^,^^104^ Patient-reported adherence measures (PRAMs) can be utilized to assess inhaler usage, though these tools should be refined based on clinical guidelines to ensure optimal accessibility and reliability.^105^

For managing asthma attacks, treatments such as oral corticosteroid (OCS),^21^ salbutamol sulfate,^106^ and omalizumab are highly effective, especially in children. While preseason omalizumab treatment serves as an effective prophylaxis for school-aged children,^107^ its efficacy in reducing the seasonal asthma exacerbation predictive index (saEPI) is less pronounced.^108^ A combination of medium dose long-acting beta2-agonist (LABA) or a long-acting muscarinic antagonist (LAMA) with inhaled corticosteroid (ICS) can significantly reduce severe asthma exacerbations, as can doubling the dose of ICS.^2^ Moreover, the combination of fast-acting beta2-agonists (FABA) with ICS is clinically effective and does not lead to adverse events.^109^ It is crucial to note that the pharmacokinetics of inhaled corticosteroids are dose-dependent, necessitating careful analytical evaluation.^110^

**Fig. 2:**
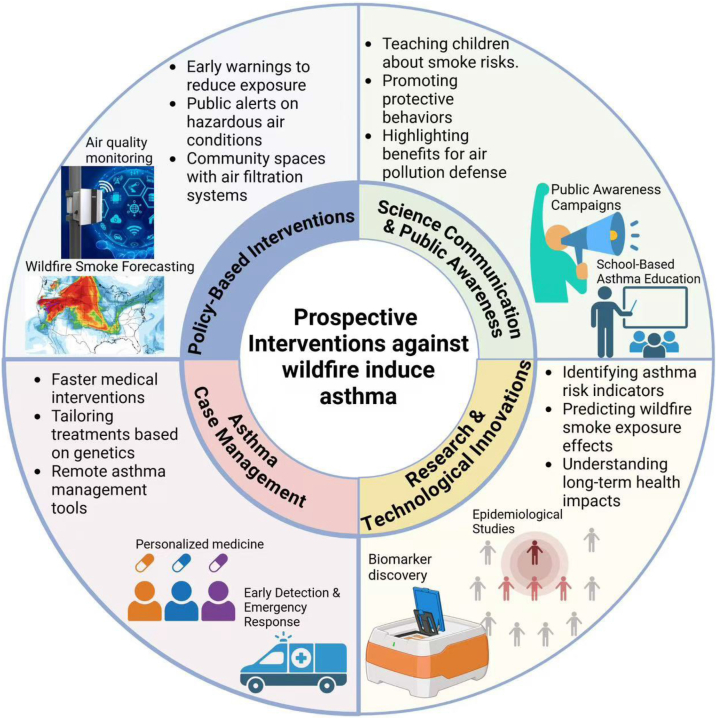
Prospective interventions against wildfire-induced asthma.

While vitamin D demonstrates efficacy in reducing emergency department visits and hospitalization rates in asthma care, its prophylactic value against childhood asthma pathogenesis remains insufficiently evidenced.^111^ Similarly, dietary interventions show limited clinically significant outcomes in asthma management according to current evidence.^112^ In precision medicine advancements, the identification of rs2660845 as an LTA4H regulatory variant interacting with montelukast response has enabled targeted development of pathophysiology-specific biomarkers and enzymatic inhibitors.^113^ Concurrently, emerging phenotyping-genotyping synergies are advancing functionalized therapeutic strategies through mechanistic stratification of airway inflammation subtypes. Telehealth technologies have been shown to be effective for asthma management,^114^ and unique recommendations from patients to physicians show promise for better care.^115^ Fig. 2.

### Recommendations on preventing wildfire-induced asthma

The advocacy of health and clean environments should include a standard evacuation strategy, particularly targeting pediatric and geriatric cohorts with pre-existing respiratory conditions. In addition, the management of wildfire-induced health risks should be an integral component as multidisciplinary and multilateral scientific policy research.

In terms of education, fundamental human physiology—such as immune responses to environmental stressors—should be demonstrated at public gatherings. Health education is not merely beneficial; it is an obligation, as neglecting this aspect can result in loss of life. Environmental issues are a collective concern that necessitate awareness across all sectors. To this end, schools, households, and communities must comprehend wildfire smoke evaluation strategies and be prepared to disseminate this information to their neighbors. This initiative could be complemented by integrated asthma counseling that emphasizes the importance of wearing face masks in polluted environments.

While the pursuit of research into the incidence of wildfires is undeniably important, it is equally crucial to explore the links between human physiology, epigenetic and genetic diversity, and vulnerability to wildfire smoke. High-throughput research techniques and AI tools should be employed in a synergistic approach to delve deeper into the health implications and to develop standardized protocols. Although there are highly skilled professionals capable of detecting and managing asthma episodes, real-time patient responses to specific medications and inhalers should be shared among physicians and across hospitals. While precision treatment for patients with wildfire-induced asthma remains a promising yet unrealized goal, telemedicine and telehealth technologies have proven to be effective medical care options.

## Conclusions

Although wildfire management experts have made significant progress in finding tangible solutions, there is a lack of multidimensional approaches not only to prevent the spread of fire, but also to prevent serious illness at the hospital level. Policies should not only focus on preventing wildfires, but also on the causes of wildfires and effective measures for case management. Moreover, the bridge in communication has disparity especially to most of the victims who cannot afford either the gadgets or the first aid expertise. Apparently, the research hub for wildfire and asthma is not deep and based mostly on observations instead of clinical trials for both short- and long-term effects. In addition, asthma from different sources may require different doses or medications, which may depend on age and severity. This review hopes to open discussions on the whole episodes of recent wildfires to draw some lessons for prospective interventions.

## Author Contributions

Xingxing Yuan wrote the manuscript, Ousman Bajinka and Liuxin Yang drew the figures, Chaofan Li and Zhuying Li revised and proofread the manuscript.

## Declaration of generative AI and AI-assisted technologies in the writing process

The author(s) declare that no generative AI was used in the creation of this manuscript.

## Abbreviations

ACS, Acute chest syndrome; AHCU, Asthma Healthcare Utilization; AI, Artificial intelligence; BCAPS, British Columbia Asthma Prediction System; BREATHE, BRief intervention to Evaluate Asthma THErapy; CHECK, Complex Children's Healthcare Coordination; COVID-19, Coronavirus Disease 2019; CRFs, Concentration-response functions; CTRP, C1q TNF-related protein; DASH, Dietary Approaches to Stop Hypertension; ERIC, Expert Recommendations for Implementing Change; FABA, Fast-acting beta2-agonists; FEV1, Forced expiratory volume in 1 s; FGF19, Fibroblast growth factor 19; FRAP, Ferric Reducing Antioxidant Power; GHGs, Greenhouse gases; Gzm A/B/H, Granzymes A, B, and H; HLCA, Human Lung Cell Atlas; ICS, Inhaled corticosteroid; IL-16, Interleukin-16; KIR3DL1, Killer cell immunoglobulin-like receptor 3DL1; LABA, Long-acting beta2-agonist; LAMA, Long-acting muscarinic antagonist; MacLEAP, Engulfed Carbon Particles; MDA, Malondialdehyde; MDI, Metered-dose inhaler; NBN, Nibrin; NO, Nitric oxide; NOE, N. officinale hydroalcoholic extract; PARP1, poly (ADP-ribose) polymerase 1; PCO, Protein Carbonyl; PFTs, Pulmonary function tests; PM, Particulate matter; PRAMs, Patient-reported adherence measures; RCT, Randomized Controlled Trial; RE-AIM, Reach Effectiveness, Adoption, Implementation, Maintenance; saEPI, seasonal asthma exacerbation predictive index; SDM, Shared decision making; SLIT, Sublingual immunotherapy; TGF-α, Transforming growth factor α; Th, T-helper; TLC, Total lung capacity; TRPV1/4, Transient receptor potential vanilloid 1/4.

## Conflicts of interest

The authors declare they have no conflicts of interest.
